# Expanded and activated allogeneic NK cells are cytotoxic against B-chronic lymphocytic leukemia (B-CLL) cells with sporadic cases of resistance

**DOI:** 10.1038/s41598-020-76051-z

**Published:** 2020-11-10

**Authors:** Tania Calvo, Chantal Reina-Ortiz, David Giraldos, María Gascón, Daniel Woods, Judit Asenjo, Joaquín Marco-Brualla, Gemma Azaceta, Isabel Izquierdo, Luis Palomera, Diego Sánchez-Martínez, Isabel Marzo, Javier Naval, Carlos Vilches, Martín Villalba, Alberto Anel

**Affiliations:** 1 Apoptosis, Immunity & Cancer Group, University of Zaragoza and Aragón Health Research Institute (IIS Aragón), Zaragoza, Spain; 2 Immunogenetics-HLA Laboratory, Puerta de Hierro Hospital, Majadahonda, Madrid, Spain; 3 Hematology Department, Lozano Blesa Hospital, Zaragoza, Spain; 4 Hematology Department, Miguel Servet Hospital, Zaragoza, Spain; 5 IRMB, Univ Montpellier, INSERM, CNRS, Montpellier, France; 6 IRMB, CHU Montpellier, Montpellier, France; 7 Apoptosis, Immunity & Cancer Group, Department Biochemistry and Molecular and Cell Biology, Faculty of Sciences, University of Zaragoza and Aragón Health Research Institute (IIS Aragón), Campus San Francisco Sq., 50009 Zaragoza, Spain

**Keywords:** Cancer, Cell biology, Immunology

## Abstract

Adoptive transfer of allogeneic natural killer (NK) cells is becoming a credible immunotherapy for hematological malignancies. In the present work, using an optimized expansion/activation protocol of human NK cells, we generate expanded NK cells (eNK) with increased expression of CD56 and NKp44, while maintaining that of CD16. These eNK cells exerted significant cytotoxicity against cells from 34 B-CLL patients, with only 1 sample exhibiting resistance. This sporadic resistance did not correlate with match between KIR ligands expressed by the eNK cells and the leukemic cells, while cells with match resulted sensitive to eNK cells. This suggests that KIR mismatch is not relevant when expanded NK cells are used as effectors. In addition, we found two examples of de novo resistance to eNK cell cytotoxicity during the clinical course of the disease. Resistance correlated with KIR-ligand match in one of the patients, but not in the other, and was associated with a significant increase in PD-L1 expression in the cells from both patients. Treatment of one of these patients with idelalisib correlated with the loss of PD-L1 expression and with re-sensitization to eNK cytotoxicity. We confirmed the idelalisib-induced decrease in PD-L1 expression in the B-CLL cell line Mec1 and in cultured cells from B-CLL patients. As a main conclusion, our results reinforce the feasibility of using expanded and activated allogeneic NK cells in the treatment of B-CLL.

## Introduction

Although the immune system can prevent the development of tumors, through a process known as anti-tumor immune surveillance, many cancers are able to evade this surveillance. This is achieved through multiple pathways such as the production of immunosuppressive cytokines, the induction of regulatory T cells and the interference with tumor antigen presentation to cytotoxic T lymphocytes (CTL)^1^. One of the advantages of T cell responses, their specificity, can also be a limitation if tumor cells develop strategies to hide tumor-specific antigen expression^2^. This limitation is not shared by NK cell responses, as they are not antigen-specific. Certain immune evasion mechanisms used by tumor cells to avoid attack by CTL are ineffective against NK cells^3^. Although they differ in antigen specificity, NK cells and CTLs share the same effector mechanisms to efficiently kill tumor cells: perforin-mediated granzyme B delivery inside target cells and death ligand-induced apoptosis, namely FasL and TRAIL^4^. Therefore, advances in the understanding of NK cell biology and function make them a powerful tool for new immunotherapies^5^, some of which are currently in clinical trials^6^.

NK cells have been tested in selected patients with aggressive or high-risk hematological cancers. The incompatibility between HLA-I molecules expressed by the tumor and the inhibitory receptors (KIR) of the donor's NK cells (mismatch) improves clinical results^7,8^ and the allogeneic NK response observed in pioneering studies is beneficial and seemed safe^8–10^. Additionally, the combination of the NK cell response with use of anti-tumor antibodies, through antibody-dependent cellular cytotoxicity (ADCC), offers therapeutic opportunities yet to be explored^11^. In previous clinical studies, NK cells transferred to patients, always respecting the incompatibility between their KIR and the HLA-I expressed by the tumors, were neither activated nor expanded^9,10,12–14^. However, recently, phase I/II clinical studies have been performed using NK cells expanded through different approaches, on multiple myeloma and acute myeloid leukemia (AML), including pediatric patients^15–18^. Other pertinent clinical trials are currently ongoing^19^. The importance of KIR mismatch in expanded NK cells is yet unknown.

B-cell chronic lymphocytic leukemia (B-CLL) is the most common leukemia in adults in the Western world and is characterized by the accumulation of mature B-lymphocytes in peripheral blood, bone marrow and secondary lymphoid organs. The leukemia cells express a variety of proteins of the Bcl-2 family that favor the inhibition of apoptosis, which, together with the interaction with the cellular microenvironment and the release of cytokines, results in the accumulation of B-CLL cells in several organs^20^. Alkylating drug-based therapies, alone or in association with corticosteroids or anti-CD20 antibodies, such as rituximab, have been the first line of B-CLL treatment for decades, as well as purine analogs. However, chemotherapeutic treatments may impair antitumor immune responses due to their immunosuppressive side effects^20,21^. Owing to the intrinsic heterogeneity of B-CLL, there is still a substantial percentage of patients with unfavorable evolution, particularly those that present mutated p53 or 17p deletion^22^. Despite advances in treatment, the 5-year mortality rate in B-CLL patients is highly variable, and patients with high-risk features still show low rates of survival^22^. Hence, new and more efficient treatments are needed.

In patients with B-CLL, the total number of NK cells in peripheral blood is increased, but they exhibit defective cytotoxic activity. Culturing NK cells with cytokines such as IL-2 and IL-15 can stimulate this activity^23^. The introduction of properly activated and expanded allogeneic NK cells, as indicated above, for the adoptive therapy of B-CLL is worth further exploration.

The immune checkpoints refer to inhibitory pathways that modulate the duration and amplitude of the physiological immune response. One of the mechanisms of immune suppression developed in cancer is the induction of these control points on the surface of activated T cells, CTLA-4 and PD-1 being the two most studied^24^. PD-1 is a receptor member of the immunoglobulin superfamily present in activated T cells. Together with its ligands, PD-L1 and PD-L2, it has an important function in the regulation of immune responses^25^. Therefore, blocking this receptor is among the most promising approaches to therapeutic anti-tumor immunity^26^. The use of anti-PD1 blocking antibodies such as pembrolizumab and nivolumab has become a first-line treatment in tumors with poor prognosis^27^. Some reports indicate that PD-L1 expression in B-CLL patients could be a negative prognostic marker, related to an exhausted phenotype in T cells^28–30^. Moreover, PD-L1 expression in tumor cells results in functional NK cell impairment^31,32^. However, no information is available on the regulation of NK cell function by PD-1 in B-CLL patients.

In this work, using an optimized protocol for expansion and activation of NK cells from healthy adult donors, we tested the expanded NK cells on samples from 35 B-CLL patients. The initial 30-patient cohort included patients at different stages of the disease, either previously treated or untreated (see Suppl. Table I). In some cases, we obtained samples from the same patients at different times during the course of the disease. This follow-up allowed for the detection of de novo resistances to eNK treatment. We undertook studies to determine the molecular basis for, and possible treatments to reverse, these resistances.

## Materials and methods

### NK cells and cells from B-CLL patients

NK cells were generated from PBMCs of healthy donors obtained from leukopaks provided by the Blood and Tissue Bank of Aragón. Cells from B-CLL patients were obtained by the hematologists involved in the study.

### NK cell expansion protocols

PBMC were isolated from leukopaks by Ficoll-Paque (Sigma) density centrifugation. Partial T cell depletion was then performed by using anti-CD3 mAb bound to magnetic beads and MACS immunomagnetic negative isolation (Miltenyi Biotec). Then, 50 × 10^6^ cells were cultured at 2 × 10^6^ cells/ml in the presence of 25 IU/ml of IL-15 and 100 IU/ml of IL-2; or with 25 IU/ml of IL-15, 100 IU/ml of IL-2 and 100 IU/ml of IFN-α. In both protocols, cells were cultured in the presence of the HLA-I negative, EBV^+^ lymphoblastoid B cell line 721.221^33^ at a 10:1 ratio, previously treated with mitomycin C to prevent their proliferation. These cultures were maintained for 20 days, with changes of medium to add fresh cytokines, and with the addition of feeder cells every 5 days. Culture viability and NK cell expansion, defined as CD3^−^ CD56^+^ by flow cytometry, was also determined. At the end of the expansion period, NK cells were isolated by positive selection using anti-CD56 magnetic beads (Miltenyi Biotec). Purity and viability of isolated NK was always 95% or higher.

### Phenotyping of expanded NK cells (eNK cells)

Expression levels of the most relevant activating and inhibitory NK cell receptors were determined in NK cells at day 0 and day 20 of expansion by flow cytometry using PE-labelled mAb. The expression of activating NCR NKp30, NKp44 and NKp46 was determined using mAb from Beckman-Coulter, clones Z25, Z231 and BAB281, respectively. NKG2D expression was determined with the clone 1D11 mAb from BD, CD16 with the clone VEP13 mAb from Miltenyi Biotec, and DNAM-1 using the clone #102511 mAb from R&D Systems. The expression of the inhibitory receptors NKG2A and ILT2 was determined using mAb from Beckman-Coulter, clones Z199 and A07408, respectively. PD-1 expression was also analyzed using an anti-PD1 mAb conjugated with FITC from Biolegend (clone EH12.2H7).

### Study of the match or mismatch bewteen eNK cells and B-CLL cells

The extraction of genomic DNA was carried out using DNAzol (MRC). The analysis of the KIR epitopes in HLA class I genes in NK cells and cells from B-CLL patients was carried out by PCR with sequence-specific primers as indicated in Sánchez-Martínez et al.^34^.

### Cytotoxic assays of eNK cells on cells from B-CLL patients

eNK cells were isolated between day 15 and 22 of expansion by positive selection using anti-CD56 magnetic beads (Miltenyi Biotec) and used for cytotoxicity assays against cell lines or cells from B-CLL patients. Cells from B-CLL patients were obtained by Ficoll-Paque density centrifugation and cultured for 2 h in complete medium with 100 IU/ml IL-4 to improve viability. All patients exhibited more than 85% of leukemic blasts in blood, so they were not separated. Purified eNK cells were labeled with 1 μM Cell Tracker Green (CTG) (Invitrogen) and mixed with target cells at a 5:1 effector to target ratio. After incubating for 4 h at 37 °C, DNA damage was measured in the target population (CTG negative cells) by flow cytometry using 7AAD (Immunostep) labeling. See Supplemental Fig. I for an example of the method using the B-CLL cell line Mec-1 as target.

### Analysis of PDL-1 expression in B-CLL cells

PD-L1 expression was analyzed on the surface of B-CLL cells from patients or on the B-CLL cell line Mec-1 using the anti-PDL1 mAb conjugated with PE (clone 10F.9G2, BioLegend) and flow cytometry analysis.

### Statistical analysis

Differences between the percentages of NK cells expressing specific surface receptors upon expansion were assessed using the Student’s t test. In the case of cytotoxicity assays, we used the one-Way ANOVA Tukey test.

Differences were considered statistically significant at *P* < 0.05.

### Ethical statement

All NK cells used were generated from PBMC of healthy donors obtained from leukopaks provided by the Blood and Tissue Bank of Aragón, under the permission of the Clinical Research Ethical Committee from Aragón (CEICA) (Ref. PI16/0129). Cells from B-CLL patients were obtained by the hematologists involved in the study, with the corresponding permission of the CEICA, reference number PI13/0146, and all patients signed an authorized informed consent. The involvement with human subjects complies with the Declaration of Helsinki.

## Results

### Expansion protocols and phenotype of expanded NK cells (eNK)

**Figure 1:**
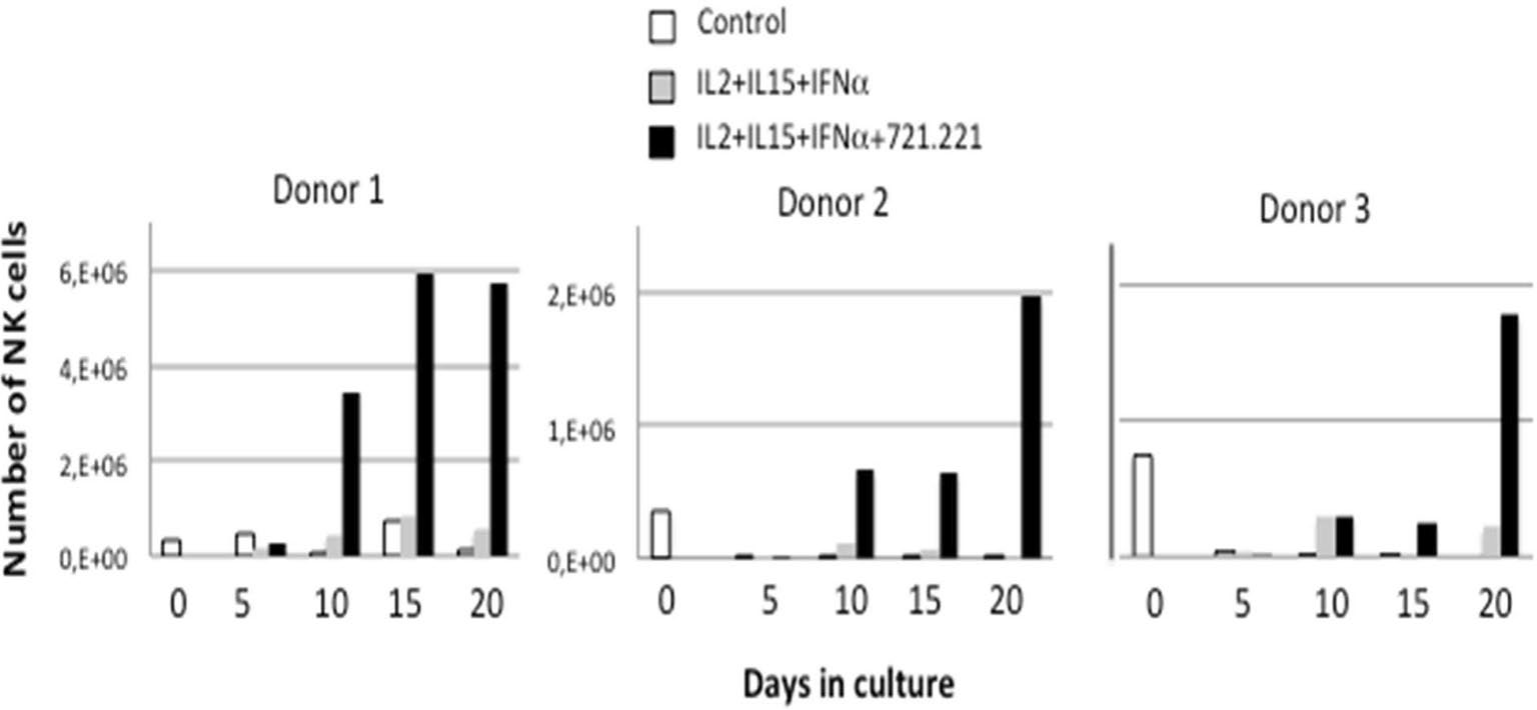
Expansion of NK cells is dependent on the presence of feeder cells. Total PBMC from three different donors were placed in culture at 2 × 10^6^ cells/ml in complete medium (Control, white bars), or in medium supplemented with 100 IU/ml IL-2, 25 IU/ml IL-15 and 100 IU/ml of IFN-α in the presence (black bars) or absence (grey bars) of 721.221 feeder cells previously treated with mitomycin C. At the times indicated, the percentage of CD3^-^CD56^+^ cells was determined by flow cytometry, viable cells counted by Trypan blue exclusion and the total number of NK cells calculated. At day 5, 10 and 15, fresh medium and cytokines and new feeder cells were added and total cell density adjusted around 2 × 10^6^ cells/ml.

In previous works, we developed a protocol for human NK cell activation using a 5-day stimulation of PBMC in the presence of lymphoblastoid cell lines transformed with the Epstein Bar virus (EBV)^34,35^, following the pioneering work of Perussia et al.^36^. Subsequently, we developed NK cell expansion protocols from umbilical cord blood^11^, which were similar to a previously reported protocol^37^. A further optimization of these expansion protocols was undertaken using NK cells from healthy donors and the HLA-I negative EBV^+^ lymphoblastoid cell line (LCL) 721.221^33^ as feeder, to avoid any inhibitory KIR signaling during expansion. Two cytokine cocktails were compared during the expansion protocol, always in the presence of the feeder cells: IL-2 + IL-15 or IL-2 + IL-15 + IFN-α. The inclusion of IFN-α is justified as this stimulatory cytokine increases cytotoxic potential in mouse NK cells in comparison with IL-15 that was instead implicated in the maintenance of viability^38^. Figure 1 shows how the presence of the feeder cells was necessary for the efficient expansion of NK cells. As expansion rates were reduced by the presence of T cells, they were partially depleted from PBMC before beginning the cultures. This allowed for a consistently greater expansion rate of NK cells, which is indicated in Supplemental Table II for the 10 donors whose cells were used in the following cytotoxicity tests. The inclusion of IFN-α did not improve the expansion rate, being the combination of IL2 + IL15 enough to support NK cell expansion.

After the 20-day expansion, the NK cell population increased in percentage and in total number, along with upregulated levels of surface CD56 expression (see Supplemental Fig. 2). The high percentage of NK cells expressing CD16, NKG2D, DNAM-1 and NKp46 in unstimulated cells was maintained in eNK cells (Supplemental Fig. 3). The low percentage of unstimulated NK cells expressing NKp44 was significantly increased in eNK cells. An average increase in the percentage of NKp30-expressing cells was observed, but it was not statistically significant. Regarding inhibitory receptor expression, the percentage of NKG2A- or ILT2-expressing cells was variable in unstimulated cells, and, although a tendency to increase was observed upon expansion, it was not statistically significant. A significant effect on the final phenotype whether IFN-α was included in the expansion protocol or not was not observed.

### Cytotoxicity of eNK cells

As shown in Suppl. Fig. 1, we first tested eNK cells in 4 h assays on the B-CLL-like cell line Mec1 and obtained 60% of specific cytotoxicity. We also tested eNK cells on the HLA-I negative erythroleukemia K562 and on the HLA-I positive acute lymphocytic leukemia cell line Jurkat, reaching specific cytotoxicity nearing 70% at the 9:1 E.T ratio in both cases (Suppl. Fig. 4A). We compared the cytotoxicity of NK cells activated for 5 or 20 days on K562 cells and found that, although cells activated for 5 days were cytotoxic, the level of cytotoxicity exerted by eNK cells was higher, especially at 3:1 or 9:1 effector to target ratios (Suppl. Fig. 4B). Then, we tested non activated NK cells (Control) or eNK cells obtained in the presence of IL2 + IL15 and in the absence (IL) or presence of IFN-α (IL + IFN) on Jurkat cells overexpressing Bcl-x_L_ (Jurkat-Bcl-x_L_). We observed that non-activated NK cells exerted less than 10% of specific cytotoxicity, while eNK cells induced between 35 and 60% depending on the donor (Suppl. Fig. 4C). Again, IFN-α did not significantly increase cytotoxicity.

Once the cytotoxic potential of eNK cells was ascertained, we were able to test them against cells from 30 B-CLL patients. The clinical data at the time of sampling are depicted in Suppl. Table I. This cohort included patients at different stages of the disease, either previously treated or untreated. Standard diagnosis protocols were followed in all the patients. In certain patients, genetic analysis of specific risk factors was also performed (see Suppl. Table I).

**Figure 2:**
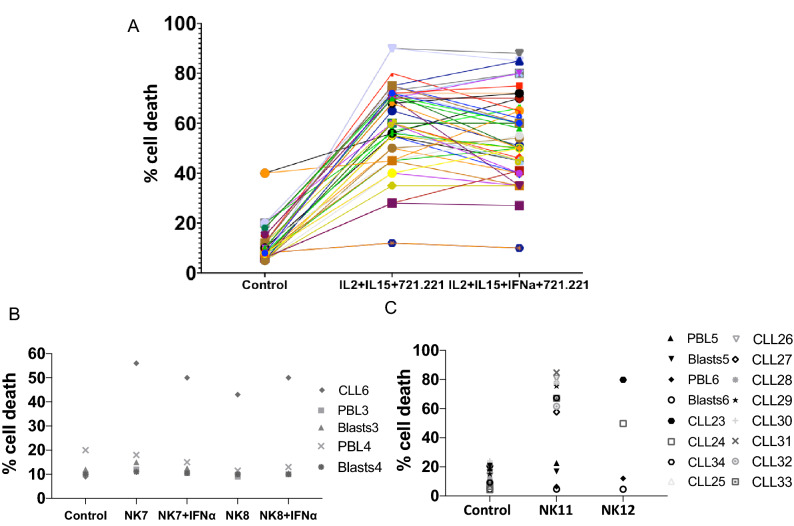
Cytotoxicity of eNK cells on cells from B-CLL patients. (**A**) Cytotoxicity of eNK cells from the 10 donors shown in Suppl. Table II and expanded in the presence (IL2 + IL15 + IFNα + 721.221) or in the absence of IFN-α(IL2 + IL15 + 721.221) on cells from 23 patients of B-CLL. Cytotoxicity tests were conducted during 4 h at a 5:1 E:T ratio. Data show the percentage of cell death in the target population estimated by 7-ADD labeling. The Control points show 7-AAD labeling of leukemic cells alone after the 4 h incubation in complete medium. (**B**) Cytotoxicity of NK7 and NK8 eNK cells, expanded in the presence (+ IFNα) or in the absence of IFN-α on cells from B-CLL patient 6 or on PBL or T cell blasts from two unrelated donors. (**C**) Cytotoxicity of NK11 and NK12 eNK cells, expanded in the presence of IL2 + IL15, but in the absence of IFN-α, on cells from B-CLL patients CLL23 to CLL34, or on PBL or T cell blasts from two additional unrelated donors. Cytotoxicity tests were conducted during 4 h at a 5:1 E:T ratio and data show the percentage of cell death in the target population estimated by 7-ADD labeling. The “Control” points show 7-ADD labeling of target cells alone after the 4 h incubation in complete medium.

In 7 samples, basal cell death was higher than 50%, precluding their use in the cytotoxicity experiments. Data presented in Fig. 2A shows how eNK cells exerted significant cytotoxicity against cells from 22 B-CLL patients, whereas only 1 were resistant, patient 18 (see Table 2). Patient 18 cells’ were the only ones that showed less than 25% specific cell death induced by eNK cells. This result was obtained when testing eNK cells from the 10 donors which expansion rates are depicted in Supplemental Table II. Basal cell death averaged 11% and cytotoxicity exerted by eNK cells on leukemic cells was variable, in some cases more than 80%. On average, cell death was 58%, significantly higher than basal values, and represented a mean of 47% specific cell death induced by eNK. Addition of IFN-α during expansion did not substantially affect the cytotoxic potential of eNK cells on cells from B-CLL patients, averaging 54% (Fig. 2A). The increase in cytotoxicity was statistically significant in both types of expansion, using a Tukey non-parametric analysis, with P = 0.001 in both cases.

In order to ascertain their specificity against tumor cells, we also tested the cytotoxicity of 2 eNK cells (NK7 and NK8) used in the cytotoxicity assays shown in Fig. 2A and two additional donors (NK11 and NK12), on freshly isolated PBMC or T cell blasts from 4 unrelated healthy donors (Fig. 2B,C). The T cell blasts were obtained through PHA stimulation in the presence of IL-2 during 5 days. The cytotoxicity of the eNK cells on normal PBMC and on T-cell blasts was low (Fig. 2B,C). Importantly, the eNK cells exerted substantial cytotoxicity against cells from B-CLL patient 6 (CLL6; Fig. 2B) and on cells from 12 additional B-CLL patients (from CLL23 to CLL34; Fig. 2C). This clearly shows that eNK cytotoxicity mainly targets transformed cells.

### Analysis of the KIR-epitope match between eNK and B-CLL cells

The sporadic resistance observed in leukemic cells from patient 18 could be due to the match between KIRs expressed by eNK cells and HLA-I expressed by the leukemic cells. The inhibitory KIRs 2DL2/3, 2DL1, 3DL1 and 3DL2 recognize the HLA class I epitopes C1, C2, Bw4 and the A3/A11 alleles, respectively^39,40^. When a target cell lacks one or more of the allotypes present in an NK-cell donor (‘KIR-ligand mismatch’), allogeneic NK-cell reactivity can be expected. KIR ligands in DNA from 22 of the B-CLL patients and from 7 of the 10 eNK with which cytotoxicity was assayed in Figs. 2A,B were genotyped. Unfortunately, we could not obtain enough genomic DNA from NK1, NK2 and NK8, indicated as N.D in Tables 1 and 2. In most of the cases, there was a mismatch between eNK cells and cells from B-CLL patients, as shown in Table 2, and those B-CLL were sensitive to eNK cytotoxicity. However, although leukemic cells from patient 18 were also mismatched with the effector cell ligands, they were resistant to cytotoxicity exerted by NK9 and NK10. Conversely, cells from patients 3 and 5 had matched KIR epitopes with their effector cells and were also sensitive to cytotoxicity exerted by NK3 and NK4 (Table 2).

**Table 1:** Expression of the C1, C2, Bw4 and A3/A11 HLA class I epitopes in B-CLL patients and in NK cells used in the cytotoxicity assays shown in Fig. 2A.

Sample	C1	C2	Bw4	A3/A11
CLL1	+	−	+	−
CLL2	+	−	+	−
CLL3	+	+	+	N.D
CLL4	+	+	+	−
CLL5	+	+	+	−
CLL6	+	−	+	−
CLL7	+	+	+	−
CLL8	+	−	+	−
CLL9	+	−	+	+
CLL10	+	−	−	+
CLL11	+	−	+	−
CLL12	+	−	+	−
CLL13	−	+	+	+
CLL14	+	−	+	−
CLL15	+	−	+	−
CLL16	+	−	+	−
CLL17	+	−	N.D	−
CLL18	+	−	+	+
CLL19	+	−	+	−
CLL20	+	−	−	−
CLL21	N.D	N.D	N.D	−
CLL22	+	+	+	−
NK1; NK2; NK8	N.D	N.D	N.D	N.D
NK3	+	+	−	−
NK4	+	+	+	−
NK5	+	+	−	+
NK6	+	+	N.D	N.D
NK7	+	−	+	+
NK9	+	+	+	−
NK10	+	+	+	−

**Table 2:** Determination of the match or mismatch between HLA class I epitopes in B-CLL patients and in NK cells used in the cytotoxicity assays shown in Fig. 2A.

Combination	Match	Resistance
NK1 or NK2/CLL1	N.D	No
NK1 or NK2/CLL2	N.D	No
NK1 or NK2/CLL4	N.D	No
NK1 or NK2/CLL7	N.D	No
NK1 or NK2/CLL8	N.D	No
NK1 or NK2/CLL9	N.D	No
NK1 or NK2/CLL10	N.D	No
NK3 or NK4/CLL1	No	No
NK3 or NK4/CLL3	**YES**	No
NK3 or NK4/CLL5	**YES**	No
NK3 or NK4/CLL11	No	No
NK3 or NK4/CLL12	No	No
NK3 or NK4/CLL13	No	No
NK5 or NK6/CLL14	No	No
NK5 or NK6/CLL15	No	No
NK5 or NK6/CLL16	No	No
NK5 or NK6/CLL17	No	No
NK7/CLL6	No	No
NK9 or NK10/CLL19	No	No
NK9 or NK10/CLL20	No	No
NK9 or NK10/CLL21	No	No
NK9 or NK10/2nd CLL5	**YES**	**YES**
NK9 or NK10/2nd CLL8	No	**YES**
NK9 or NK10/CLL18	No	**YES**

Bold indicates the situations in which a match or a resistance could be detected.

*N.D.* not determined.

### Sporadic development of resistances correlates with high PD-L1 expression

**Figure 3:**
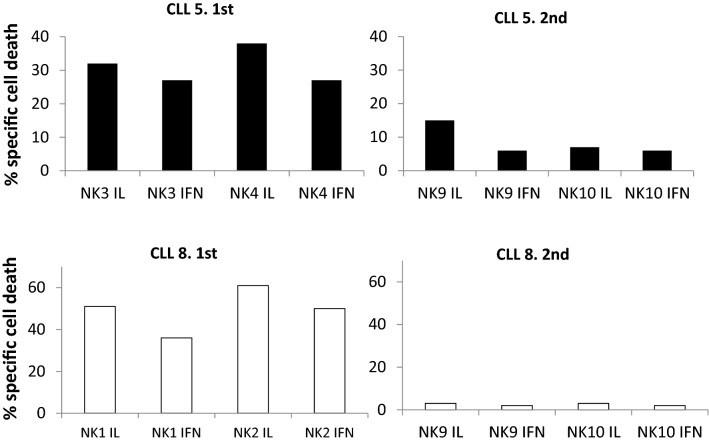
Development of resistance to eNK cells in two B-CLL patients. Upper panels: NK3 and NK4 cells expanded in the presence (IFN) or in the absence of IFN-α were tested at a 5:1 E:T ratio against cells from B-CLL patient 5 at the first time of sample acquisition. Some months later, cells from the same patient were obtained for a 2nd time and confronted with NK9 and NK10. Lower panels: NK1 and NK2 cells expanded in the presence (IFN) or in the absence of IFN-α were tested at a 5:1 E:T ratio against cells from B-CLL patient 8 at the first time of sample acquisition. Some months later, cells from the same patient were obtained for a 2nd time and confronted with NK9 and NK10. Cell death was tested by 7-AAD labeling. Results are shown as the percentage of specific cell death induced, after subtracting basal leukemic cell death, which was never higher than 15%.

In two patients (CLL5 and CLL8), samples were obtained at different stages of the disease, separated temporally by several months. CLL5 cells were sensitive to NK3 and NK4 at the time of the 1^st^ sample acquisition, but some months later, they showed resistance to NK9 and NK10 (Fig. 3, upper panels). CLL8 cells were sensitive to NK1 and NK2, but again showed almost complete resistance to NK9 and NK10 some months later (Fig. 3, lower panels). This was not due to a deficient activation of NK9 and NK10 as these eNK cells were effective against leukemic cells from patients 19, 20 and 21 (44%, 45% and 35% of specific cytotoxicity, respectively; see Table 2). Unfortunately, experiments could not be repeated with eNK cells from NK1, NK2, NK3 and NK4 on patient samples at that moment of disease progression as the entire expanded population was spent in the experiments performed on the different B-CLL patients tested in the first assays and shown in Fig. 2. In addition, the law protects the identity of the volunteer donors of the Blood Bank and obtaining a second identical sample was impossible.

The clinical data of CLL5 and CLL8 was analyzed to identify possible common features. CLL5 was 75 years-old at first sampling and, after having undergone 6 R-COP cycles and one R-Benda cycle was in partial remission (Suppl. Table III). The patient exhibited a 13q deletion in heterozygosis, a factor that in principle is associated with relative low risk^41^, 70% positivity for CD38, and 10% positivity for ZAP70. From the first to the second analysis, CLL5 had progressed from stage IA to IV-C and was under treatment with R-Benda at the time of the second sample. CLL8 was 89-years old at the time the first sample was obtained. This patient had an indolent disease, which did not evolve from stage 0, and was without any treatment, conditions that continued at the time of the second sample (Suppl. Table III). From this, eNK resistance development could be associated with a disease worsening in CLL5 but not in the case of CLL8. Common clinical features were not found between these patients.

Regarding a possible match between eNK cells and leukemic cells, KIR epitopes expressed by cells from CLL5 were indeed in match with the ligands expressed by NK9 and NK10, and, in this case, the match correlated with the resistance to cytotoxicity. For CLL8, the leukemic cells were sensitive to cytotoxicity exerted by NK1 and NK2, although the HLA-I haplotypes of NK cell donors could not be analyzed. However, cells from this patient were resistant to cytotoxicity exerted by NK9 and NK10, and no match was observed in that case (Table 2). Resistance to eNK cell cytotoxicity correlated with match in one of the patients, but not in the other, indicating that, although match could contribute to the final outcome, other intrinsic factors of leukemic cells should also account for resistance to eNK cells.

**Figure 4:**
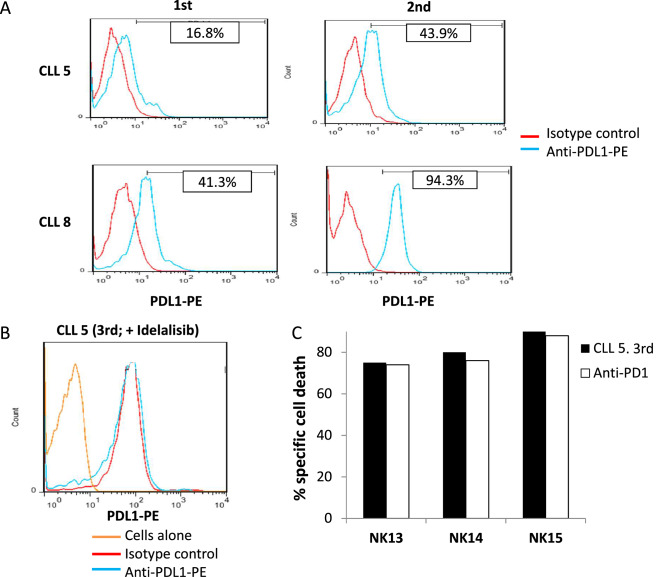
Expression of PDL-1 in leukemic cells sensitive and resistant to eNK cells. Effect of idelalisib treatment. (**A**) The expression of PDL-1 on the surface of cells from B-CLL patients 5 and 8 at the time when they were first tested against eNK cells, or when they showed resistance towards eNK cells at the 2nd time of sample acquisition, was analyzed by flow cytometry using an specific mAb conjugated with PE (blue histograms). Red histograms show the labeling on the same cells using an irrelevant mouse antibody of the same isotype and labeled with the same fluorophore. Numbers on the histograms show the percentage of cells positive for the labeling in each case. (**B**) PDL-1 expression on the surface of cells from B-CLL patient 5 at the 3rd time of sample acquisition, after treatment with idelalisib, was analyzed by flow cytometry using a specific mAb conjugated with PE (blue histogram). Red histogram shows the labeling on the same cells using an irrelevant antibody of the same isotype and labeled with the same fluorophore. Orange histogram shows the signal obtained with the same cells without labeling. (**C**) NK13, NK14 and NK15 cells were expanded using 721.221 cells as feeders in the presence of IL2 + IL15 and tested in 4 h assays at a 5:1 E:T ratio against cells from B-CLL patient 5 at the 3^rd^ time of sample acquisition, after treatment with idelalisib, in the absence (black bars) or in the presence of 10 μg/ml of the anti-PD-1 mAb pembrolizumab (white bars). Leukemic cell death was tested by 7-AAD labelling. Results are shown as the percentage of cell death, after subtracting basal leukemic cell death, which was never higher than 10%.

Remarkably, both the percentage of cells positive for PDL-1 expression and the mean expression level (MFI) increased in the two resistant samples as compared to the sensitive patient samples (Fig. 4A).

### Effect of idelalisib treatment on PD-L1 expression and on sensitivity to eNK cells

A third sample from CLL5 was obtained after the resistance to eNK treatment was developed. Between the second and third sampling, the patient was treated in clinic with the PI3Kδ inhibitor idelalisib, which is used in B-CLL treatment^42^. Remarkably, PD-L1 expression was lost in the leukemic cells of the patient after treatment with idelalisib (compare Fig. 4B with Fig. 4A, upper panels). Unfortunately, none of the eNKs used on the 1st and 2nd samples from this patient were available, as the 3^rd^ sample was taken almost 2 years later.

**Figure 5:**
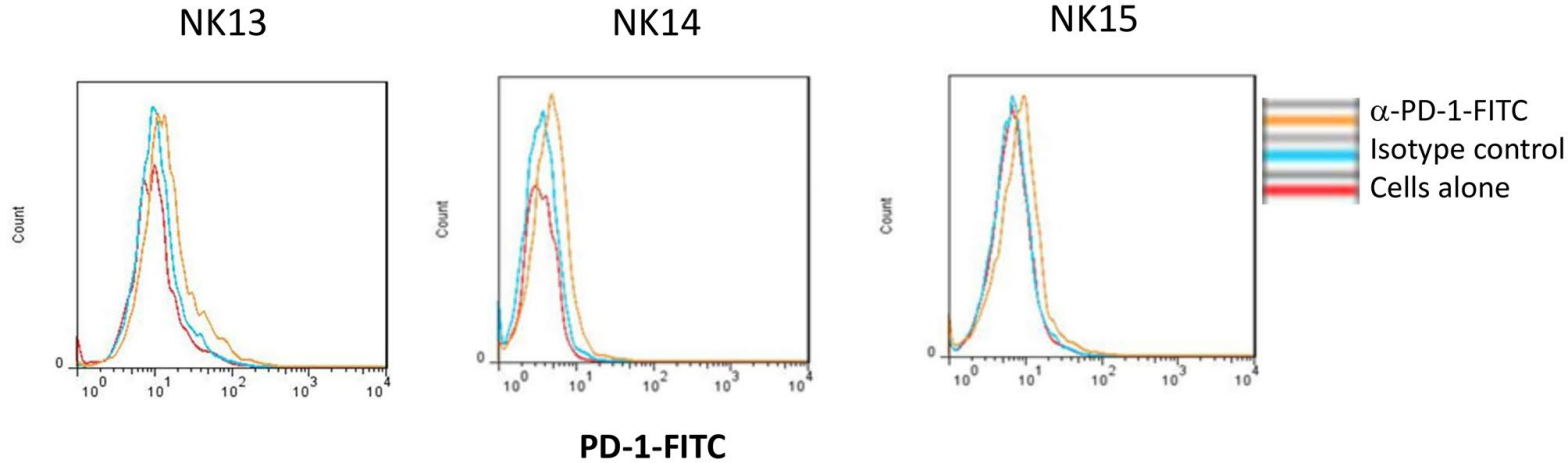
PD-1 expression in eNK cells. PD-1 expression on NK cells after 20 days of expansion was analyzed by flow cytometry on CD56^+^ cells using a specific anti-PD-1 mAb on the indicated 3 different donors, NK13, NK14 and NK15. Red histograms show the labeling of cells alone, blue histograms the labelling obtained using an irrelevant antibody of the same isotype and labeled with the same fluorophore and orange histograms the specific PD-1 labeling.

eNK cells were generated from 3 new donors (NK13, NK14 and NK15), also obtained from the Blood and Tissue Bank of Aragón, and tested against the third sample of CLL5. This was perfromed in the presence of the blocking anti-PD-1 mAb pembrolizumab. As shown in Fig. 4C, B-CLL cells from CLL5 recovered the sensitivity to cytotoxicity exerted by the three eNK cells, reaching specific cytotoxicity levels of 80%, contrasting with the resistance observed in 2nd sample from this patient (see Fig. 3). The anti-PD-1 blocking mAb pembrolizumab did not further increase these high cytotoxicity levels, as expected since target cells did not express PD-L1. We also analyzed the PD-1 expression in the eNK cells from these donors, observing that they were rather negative (Fig. 5), correlating with the lack of effect of pembrolizumab. We analyzed PD-1 expression in a total of 14 eNK cell productions and we can conclude that our expansion protocol generates eNK cells with a small percentage of PD1^+^ cells in most cases: only in 4 of those 14 donors was there a population of PD1^+^ cells representing more than 30% of eNK cells.

**Figure 6:**
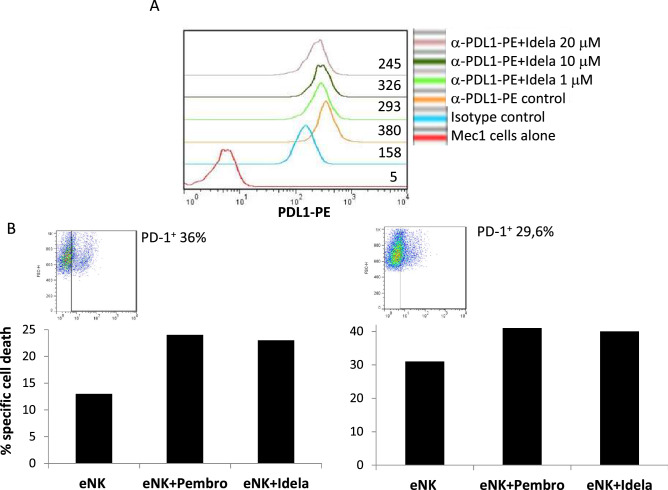
Effect of idelalisib on PDL-1 expression and on eNK cytotoxicity in the B-CLL cell line Mec-1. (**A**) The expression of PDL-1 on the surface of the B-CLL cell line Mec-1 was analyzed in the absence of idelalisib treatment (orange histogram) or after 48 h incubation with 1 μM (green histogram), 10 μM (black histogram) or 20 μM idelalisib (purple histogram). Red histogram corresponds to the labeling of the cells alone, and blue histogram shows the labeling using an irrelevant mouse antibody of the same isotype and labeled with the same fluorophore. Numbers on the histograms correspond to MFI values in each sample. (**B**) NK cells from donors 16 and 17 were expanded using 721.221 cells as feeders in the presence of IL-2 + IL-15 and tested overnight at a 1:1 E:T ratio against Mec-1 cells in the absence (eNK) or in the presence of 10 μg/ml of the anti-PD1 blocking mAb pembrolizumab (eNK + pembro), or after 48 h supplementation with 10 μM idelalisib (eNK + Idela). Mec-1 cell death was tested by annexin-V-APC labelling. The expression of PD-1 in the eNK cells used in each case is shown in the insets.

As the observation on the effect of idelalisib on PD-L1 expression could be interesting from a clinical perspective, we further studied this effect and its impact on sensitivity to eNK cells in both the B-CLL cell line Mec-1 and on cells from B-CLL patients. Mec-1 cells are positive for PD-L1 expression at the basal level (see Fig. 6A). First, we performed a dose–response between 1 and 20 μM of idelalisib on this cell line, and, although it partially inhibits cell growth (50% inhibition at the highest dose), it was not able to induce cell death in this concentration range. Next, we analyzed the effect of those doses of idelalisib on PD-L1 expression. We observed a clear reduction in PD-L1 surface expression, with a 40% reduction at 1 μM, 24% at 10 µM and peaking at 60% reduction with 20 μM (Fig. 6A). Lastly, eNK cytotoxicity was tested in either the presence of the blocking anti-PD1 mAb pembrolizumab or after 48 h supplementation with 10 μM idelalisib, arriving at a similar increase in cytotoxicity on Mec-1 cells in both cases (Fig. 6B). These experiments were performed with eNK cells from two donors (NK16 and NK17) that expressed PD-1 in at least a 30% of their population (see the insets in Fig. 6B). The tests were also performed with eNK cells with low or null PD-1 expression from other donors, and, in those cases, no effect of idelalisib or of pembrolizumab was observed (data not shown).

**Figure 7:**
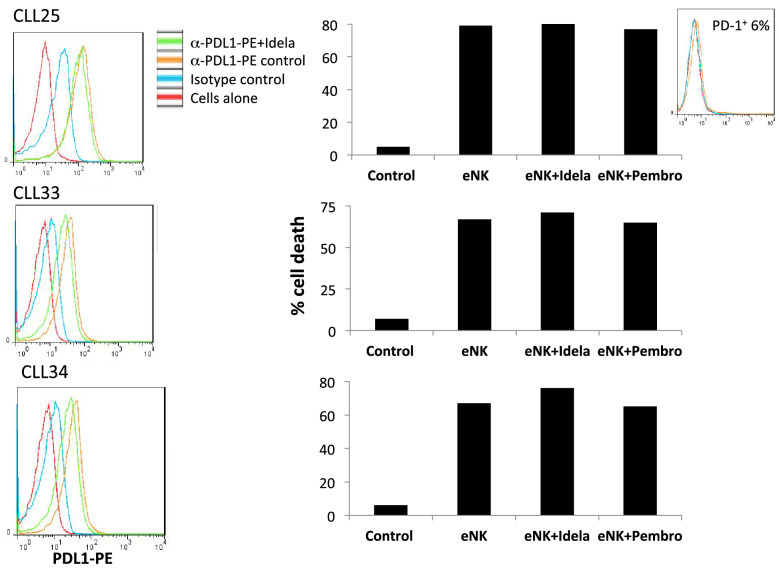
Effect of idelalisib on PDL-1 expression and on eNK cytotoxicity in cells from B-CLL patients. Left panels: The expression of PDL-1 on the surface of cells from three B-CLL patients positive for PD-L1 was analyzed in the absence of idelalisib treatment (orange histogram) or after 48 h incubation with 20 μM idelalisib (green histogram). Red histograms correspond to the labeling of the cells alone, and blue histograms show the labeling using an irrelevant mouse antibody of the same isotype and labeled with the same fluorophore. Right panels: NK18 cells were expanded using 721.221 cells as feeders in the presence of IL-2 + IL-15 and tested at a 5:1 E:T ratio for 4 h against cells from the patients after 48 h supplementation with 100 IU/ml IL-4 in the absence (eNK) or in the presence of 20 μM idelalisib (eNK + Idela). Tests were also performed in the presence of 10 μg/ml of the anti-PD1 blocking mAb pembrolizumab (eNK + pembro). Cell death was tested by 7-AAD labeling on target cells. The expression of PD-1 in the eNK cells used is shown in the inset.

PD-L1 expression and the effect of idelalisib in cells from 12 additional B-CLL patients was tested: 6 were positive for PD-L1 expression and 6 were negative. In those patients with higher PD-L1 expression, we observed how incubation with a non-toxic dose of idelalisib (10 µM) in the presence of IL-4 for 48 h resulted in a net reduction of specific PD-L1 expression of around 30% (Fig. 7, left panels). We tested the cytotoxicity of eNK cells from one additional donor (NK18) on those leukemic cells and observed that it was high at the basal level. Cytotoxicity was slightly increased by incubation with idelalisib, but this effect was not reproduced by treatment with pembrolizumab (Fig. 7, right panels). This correlates again with the almost null expression of PD-1 in eNK cells from this donor (see the inset).

## Discussion

The present work demonstrates how activation and expansion of NK cells in the presence of IL-2, IL-15 and the EBV^+^, HLA-I negative 721.221 cells clearly increases their cytotoxicity on cells from B-CLL patients. The average expansion rates obtained should allow for the treatment of one leukemic patient using eNK cells from one donor. We observed cytotoxicity in vitro in 92% of the patients’ samples tested. Meanwhile, eNK cells were not cytotoxic against PBMC or T cell blasts obtained from healthy donors. Altogether, these data reinforce the feasibility of using expanded NK cells in the treatment of B-CLL. This is especially relevant, as this approach based on NK cell activation, either alone or in combination with antibodies, is still not approved as a B-CLL treatment. Selected patients with aggressive refractory disease would especially benefit from eNK cell therapy. In some clinical contexts, clinicians used CAR T cells. This approach, however, is more expensive and has secondary effects. NK cells, in view of their low toxicity^5^, will probably have fewer undesirable effects.

The sporadic resistance observed initially in one patient did not correlate with match between the KIR ligands expressed by eNK cells and HLA-I expressed by leukemic cells. In fact, we observed a match in four cases in which eNK cells were cytotoxic. Previously we have stimulated NK cells for 5 days with LCL in the absence of cytokines^34^, in which NK cell expansion was more limited. In that study, B-CLL susceptibility significantly correlated with HLA mismatch between NK cell donor and B-CLL patient. In our present study we expanded NK cells that reached a more active status against cells from B-CLL patients and data support that, in most instances, the activation and expansion of NK cells can significantly overcome KIR match limitation.

Phenotypically, eNK cells substantially increased CD56 expression and maintained that of CD16, indicating that eNK cells could combine with therapeutic anti-tumor antibodies. The population of NKp44^+^ cells increased in eNK cells. A similar study demonstrated that the main change justifying the increase in cytotoxicity was the net increase in the level of granzyme B expression in activated NK cells. In that study, using a 5-day activation protocol in the absence of cytokines, the most significant increase in receptor expression was, as in our case, that of NKp44^35^.

We also observed two sporadic cases of de novo resistance to eNK cells during the clinical course of the disease. Both patients did not possess any common feature in either the state of the disease or in the treatment received that may have caused their resistance. A KIR match could explain resistance, but this was found in only one of the two cases. Remarkably, PD-L1 expression increased substantially in the two resistant samples from the first to the second testing.

PD-1 is an inhibitory receptor present in NK cells and activated CD4^+^ and CD8^+^ T lymphocytes involved in immunosuppression by binding to its ligands PD-L1 and PD-L2, the former showing a broader expression^25^. T cells from B-CLL patients presented defects in the formation of the immunological synapse that correlated with increased expression of PD-L1 in leukemic cells and of PD-1 in T lymphocytes^30^. B-CLL patients with the Richter transformation benefit from anti-PD-1 treatment^43^. The immunomodulatory drug lenalidomide decreases PD-L1 expression in leukemic cells and PD-1 in T cells^30^ and does not increase PD-1 in NK cells^44^. Lenalidomide and the anti-CD20 mAb obinutuzumab enhance NK cell activation markers in B cell lymphoma patients^44^. The expression of PD-1 and CTLA-4 was higher in T lymphocytes of B-CLL patients than in healthy donors of the same age^28,29^. When NK cells were expanded in the presence of an anti-PD-1 blocking antibody, NK cells had greater cytotoxicity, although this expansion protocol used an anti-CD16 antibody and IL-2, and the anti-PD1 blocking antibody was not used in cytotoxicity assays^45^. PD-L1 expression in tumor cells decreases NK cell cytotoxicity in vitro^32^. PD-1^+^ NK cells exist in humans, showing a semi-exhausted phenotype, and this population increases in patients with ovarian carcinoma^46^. Moreover, PD-1 expression mediates NK cell functional exhaustion in patients with Kaposi’s sarcoma^31^.

Patient follow-up allowed recovery of cells from one patient some months after the observed resistance. During this time, the patient had been treated with the PI3Kδ inhibitor idelalisib, which has been recently introduced in the treatment of B-CLL^42^. Remarkably, the expression of PD-L1 was lost and leukemic cells were again highly sensitive to eNK cells cytotoxicity from three different donors. This observation could have clinical interest, and although it was limited to one patient and should be interpreted with caution, we confirmed the reduction of PD-L1 expression and the increase in sensitivity to eNK cytotoxicity mediated by idelalisib in the B-CLL cell line Mec-1. The partial reduction in PD-L1 expression induced by idelalisib in cells from B-CLL patients that were initially positive for its expression was also confirmed. These data indicate that PD-L1 expression is dependent on PI3Kδ activity in B-CLL cells. Remarkably, a recent study has shown a dependence of PD-L1 expression on the PI3K/Akt signaling pathway in anaplastic large cell lymphoma^47^.

Our expansion protocol generates eNK cells negative for PD-1 expression in most cases. However, in those donors in whom a population of PD-1^+^ cells is present, it could contribute to a diminished activity on PD-L1^+^ targets.

Our present data reinforce the feasibility of using eNK cells in the treatment of B-CLL, in view of their high cytotoxicity against leukemic cells from most B-CLL patients. We propose the clinical use of eNK cells on B-CLL patients, alone or in combination with therapeutic anti-CD20 antibodies, as suggested in our recent study^11^. Additionally, although limited to two patients, our data suggest that anti-PD-1 blocking mAbs or idelalisib could improve adoptive immunotherapy with allogeneic eNK against B-CLL in especially refractory patients.

## Supplementary information

Supplementary Information.

## Data Availability

The data that support the findings of this study are available from the corresponding author upon reasonable request.

## Abbreviations

NK: Natural killer
eNK: Expanded NK cells
KIR: Killer-cell immunoglobulin-like receptors
ADCC: Antibody-dependent cell-mediated cytotoxicity
AML: Acute myeloid leukemia
B-CLL: B-cell chronic lymphocytic leukemia
CTLA-4: Cytotoxic T lymphocyte antigen 4
PD-1: Programmed death 1
PD-L1: Programmed death ligand 1
EBV: Epstein Barr virus
LCL: Lymphoblastoid cell line
HLA-I: Human leukocyte antigen class I
IL-2: Interleukin 2
IL-15: Interleukin 15
IFN-α: Interferon-α
IL-4: Interleukin 4
NKG2D: NK cell receptor group 2D
NKG2A: NK cell receptor group 2A
NKp30, NKp44, NKp46: NK cell activating receptors p30, p44 and p46, respectively
NCR: NK cell activating receptors
DNAM-1: DNAX accessory molecule 1
IL-T2: Ig-like transcript 2
R-COP: Rituximab plus cyclophosphamide, vincristine and prednisone
R-Benda: Rituximab plus bendamustine
MFI: Mean fluorescence intensity
PBMC: Peripheral blood mononuclear cells
FITC: Fluorescein isothiocyanate
PE: Phycoerythrin
APC: Allophycocyanin
7-AAD: 7-Amino actinomycin D
CTG: Cell tracker green

## References

[1] Schreiber R, Old LJ, Smyth MJ (2011). Cancer immunoediting: integrating immunity’s roles in cancer suppression and promotion. Science.

[2] Rooney M, Shukla S, Wu C, Getz G, Hacohen N (2015). Molecular and genetic properties of tumors associated with local immune cytolytic activity. Cell.

[3] Catalán E, Charni S, Jaime P, Aguiló J, Enríquez J, Naval J, Pardo J, Villalba M, Anel A (2015). MHC-I modulation due to changes in tumor cell metabolism regulates tumor sensitivity to CTL and NK cells. OncoImmunology.

[4] Martinez-Lostao L, Anel A, Pardo J (2015). How do cytotoxic lymphocytes kill cancer cells?. Clin. Cancer Res..

[5] Villalba M, Alexia C, Bellin-Robert A, Fayd'herbe de Maudave A, Gitenay D (2020). Non-genetically improving the natural cytotoxicity of natural killer (NK) cells. Front. Immunol..

[6] Morvan M, Lanier L (2016). NK cells and cancer: you can teach innate cells new tricks. Nat. Rev. Cancer.

[7] Stern M (2008). Survival after T cell-depleted haploidentical stem cell transplantation is improved using the mother as donor. Blood.

[8] Willemze R, Rodrigues CA, Labopin M, Sanz G, Michel G, Socie G, Rio B, Sirvent A, Renaud M, Madero L (2009). KIR-ligand incompatibility in the graft-versus-host direction improves outcomes after umbilical cord blood transplantation for acute leukemia. Leukemia.

[9] Miller JS, Soignier Y, Panoskaltsis-Mortari A, McNearney SA, Yun GH, Fautsch SK, McKenna D, Le C, Defor TE, Burns LJ (2005). Successful adoptive transfer and in vivo expansion of human haploidentical NK cells in patients with cancer. Blood.

[10] Rubnitz JE, Inaba H, Ribeiro RC, Pounds S, Rooney B, Bell T, Pui CH, Leung W (2010). NKAML: a pilot study to determine the safety and feasibility of haploidentical natural killer cell transplantation in childhood acute myeloid leukemia. J. Clin. Oncol..

[11] Sánchez-Martínez D, Allende-Vega N, Orecchioni S, Talarico G, Cornillon A, Vo D, Rene C, Lu Z, Krzywinska E, Anel A, Gálvez E, Pardo J (2018). Expansion of allogeneic NK cells with efficient antibody-dependent cell cytotoxicity against multiple tumors. Theranostics.

[12] Curti A, Ruggeri L, Parisi S, Bontadini A, Dan E, Motta M, Rizzi S, Trabanelli S, Ocadlikova D, Lecciso M (2016). Larger size of donor alloreactive NK cell repertoire correlates with better response to NK cell immunotherapy in elderly acute myeloid leukemia patients. Clin. Cancer Res..

[13] Nguyen S, Béziat V, Norol F, Uzunov M, Trebeden-Negre H, Azar N, Boudifa A, Bories D, Debré P (2011). Infusion of allogeneic natural killer cells in a patient with acute myeloid leukemia in relapse after haploidentical hematopoietic stem cell transplantation. Transfusion.

[14] Shi J (2008). Infusion of haplo-identical killer immunoglobulin-like receptor ligand mismatched NK cells for relapsed myeloma in the setting of autologous stem cell transplantation. Br. J. Hematol..

[15] Leivas A, Perez-Martinez A, Blanchard M, Martın-Clavero E, Fernandez L, Lahuerta J, Martinez-Lopez J (2016). Novel treatment strategy with autologous activated and expanded natural killer cells plus anti-myeloma drugs for multiple myeloma. OncoImmunology.

[16] Pérez-Martínez A (2015). A phase I/II trial of interleukin-15–stimulated natural killer cell infusion after haplo-identical stem cell transplantation for pediatric refractory solid tumors. Cytotherapy.

[17] Romee R, Rosario M, Berrien-Elliott M, Wagner J, Jewell B, Schappe T, Leong J, Abdel-Latif S, Schneider S (2016). Cytokine-induced memory-like natural killer cells exhibit enhanced responses against myeloid leukemia. Sci. Transl. Med..

[18] Szmania S, Lapteva N, Garg T, Greenway A, Lingo J, Nair B, Stone K, Woods E, Khan J, Stivers J, Panozzo S, Campana D (2015). Ex Vivo expanded natural killer cells demonstrate robust proliferation in vivo in high-risk relapsed multiple myeloma patients. J. Immunother..

[19] Childs R, Carlsten M (2015). Therapeutic approaches to enhance natural killer cell cytotoxicity against cancer: the force awakens. Nat. Rev. Drug Discov..

[20] Ghia P, Ferrari A, Caligaris-Cappio F (2007). Chronic lymphocytoc leukemia. Crit. Rev. Oncol. Hematol..

[21] Zenz T, Mertens D, Kuppers R, Dohner H, Stilgenbauer S (2010). From pathogenesis to treatment of chronic lymphocytic leukaemia. Nat. Rev. Cancer.

[22] Hallek M (2017). Chronic lymphocytic leukemia: 2017 update on diagnosis, risk stratification, and treatment. Am. J. Hematol..

[23] Sanchez C, Le Treut T, Boehrer A, Knoblauch B, Imbert J, Olive D, Costello R (2011). Natural killer cells and malignant haemopathies: a model for the interaction of cancer with innate immunity. Cancer Immunol. Immunother..

[24] Sharma P, Allison J (2015). The future of immune checkpoint therapy. Science.

[25] Boussiotis V (2016). Molecular and biochemical aspects of the PD-1 checkpoint path. N. Engl. J. Med..

[26] Pardoll D (2012). The blockade of immune checkpoints in cancer immunotherapy. Nat. Rev. Cancer.

[27] Ribas A (2012). Tumor immunotherapy directed at PD-1. N. Engl. J. Med..

[28] Brusa D, Serra S, Coscia M, Rossi D, D’Arena G, Laurenti L, Jaksic O, Fedele G, Inghirami G, Gaidano G, Malavasi F, Deaglio S (2013). The PD-1/PD-L1 axis contributes to T-cell dysfunction in chronic lymphocytic leukemia. Haematologica.

[29] Palma M, Gentilcore G, Heimersson K, Mozaffari F, Nasman-Glaser B, Young E, Rosenquist R, Hansson L, Osterborg A, Mellstedt H (2017). T cells in chronic lymphocytic leukemia display dysregulated expression of immune checkpoints and actvation markers. Haematologica.

[30] Ramsay A, Clear A, Fatah R, Gribben J (2012). Multiple inhibitory ligands induce impaired T-cell immunologic synapse function in chronic lymphocytic leukemia that can be blocked with lenalidomide: establishing a reversible immune evasion mechanism in human cancer. Blood.

[31] Beldi-Ferchiou A, Lambert M, Dogniaux S, Vély F, Vivier E, Olive D, Dupuy S, Levasseur F, Zucman D, Lebbé C, Sène D, Hivroz C (2017). PD-1 mediates functional exhaustion of activated NK cells in patients with Kaposi sarcoma. Oncotarget.

[32] Bellucci R, Martin A, Bommarito D, Wang K, Hansen S, Freeman G, Ritz J (2015). Interferon-gamma-induced activation of JAK1 and JAK2 suppresses tumor cell susceptibility to NK cells through upregulation of PD-L1 expression. OncoImmunology.

[33] Moraru M, Cañizares M, Muntasell A, de Pablo R, Lopez-Botet M, Vilches C (2012). Assessment of copy-number variation in the NKG2C receptor gene in a single-tube and characterization of a reference cell panel, using standard polymerase chain reaction. Tissue Antigens.

[34] Sánchez-Martínez D, Lanuza P, Gómez N, Muntasell A, Cisneros E, Moraru M, Azaceta G, Anel A, Martínez-Lostao L, Villalba M, Palomera L, Vilches C (2016). Activated allogeneic NK cells preferentially kill poor prognosis B-cell chronic lymphocytic leukemia cells. Front. Immunol..

[35] Sánchez-Martínez D, Azaceta G, Muntasell A, Aguiló N, Núñez D, Gálvez E, Naval J, Anel A, Palomera L, Vilches C, Marzo I, Villalba M (2015). Human NK cells activated by EBV+ lymphoblastoid cells overcome anti-apoptotic mechanisms of drug resistance in haematological cancer cells. OncoImmunology.

[36] Perussia B, Ramoni C, Anegon I, Cuturi M, Faust J, Trinchieri G (1987). Preferential proliferation of natural killer cells among peripheral blood mononuclear cells cocultured with B lymphoblastoid cell lines. Nat. Immun. Cell Growth Regul..

[37] Vasu S, Berg M, Davidson-Moncada J, Tian X, Cullis H, Childs R (2015). A novel method to expand large numbers of CD56+ natural killer cells from a minute fraction of selectively accessed cryopreserved cord blood for immunotherapy after transplantation. Cytotherapy.

[38] Comet N, Aguiló J, Rathoré M, Catalán E, Garaude J, Uzé G, Naval J, Pardo J, Villalba M, Anel A (2014). IFNα signaling through PKC-θ is essential for antitumor NK cell function. OncoImmunology.

[39] Parham P (2005). MHC class I molecules and KIRs in human history, health and survival. Nat. Rev. Immunol..

[40] Symons H, Fuchs E (2008). Hematopoietic SCT from partially HLA-mismatched (HLA-haploidentical) related donors. Bone Marrow Transplant..

[41] Van Dyke D, Shanafelt TD, Call T, Zent C, Smoley S, Rabe K, Schwager S, Sonbert J, Slager S, Kay N (2010). A comprehensive evaluation of the prognostic significance of 13q deletions in patients with B-chronic lymphocytic leukaemia. Br. J. Hematol..

[42] Brown J, Byrd J, Coutre S, Benson D, Flinn I, Wagner-Johnston N, Spurgeon S, Kahl B, Bello C, Webb H, Johnson D (2014). Idelalisib, an inhibitor of phosphatidylinositol 3-kinase p110δ, for relapsed/refractory chronic lymphocytic leukemia. Blood.

[43] Ding W, LaPlant B, Call T, Parikh S, Leis J, He R, Shanafelt T, Sinha S, Le-Rademacher J (2017). Pembrolizumab in patients with CLL and Richter transformation or with relapsed CLL. Blood.

[44] Vo D, Alexia C, Allende-Vega N, Morschhauser F, Houot R, Menard C, Tarte K, Cartron G, Villalba M (2018). NK cell activation and recovery of NK cell subsets in lymphoma patients after obinutuzumab and lenalidomide treatment. OncoImmunology.

[45] Guo Y, Feng X, Jiang Y, Shi X, Xing X, Liu X, Li N, Fadeel B, Zheng C (2016). PD1 blockade enhances cytotoxicity of in vitro expanded natural killer cells towards myeloma cells. Oncotarget.

[46] Pesce S, Greppi M, Tabellini G, Rampinelli F, Parolini S, Olive D, Moretta L, Moretta A, Marcenaro E (2017). Identification of a subset of human natural killer cells expressing high levels of programmed death 1: a phenotypic and functional characterization. J. Allergy Clin. Immunol..

[47] Zhang J, Song Z, Wang H, Lang L, Yang Y, Xiao W, Webster D, Wei W, Barta S, Kadin M, Staudt L, Nakagawa M (2019). A novel model of controlling PD-L1 expression in ALK1 anaplastic large cell lymphoma revealed by CRISPR screening. Blood.

