# Supplementary material for: Expanded and activated allogeneic NK cells are cytotoxic against B-chronic lymphocytic leukemia (B-CLL) cells with sporadic cases of resistance
Source: Sci Rep. 2020 Nov 10;10:19398. doi: 10.1038/s41598-020-76051-z (PMC7655821; doi:10.1038/s41598-020-76051-z)
Supplement: Supplementary file 1 — Supplementary Information. [file 41598_2020_76051_MOESM1_ESM.docx]

**SUPPLEMENTAL MATERIAL**

**Expanded and activated allogeneic NK cells are cytotoxic against B-chronic lymphocytic leukemia (B-CLL) cells with sporadic cases of resistance.**

**by**

**Tania Calvo, Chantal Reina-Ortiz, David Giraldos, María Gascón, Daniel Woods, Judit Asenjo, Joaquín Marco-Brualla, Gemma Azaceta, Isabel Izquierdo, Luis Palomera, Diego Sánchez-Martínez, Isabel Marzo, Javier Naval, Carlos Vilches, Martín Villalba & Alberto Anel**

**
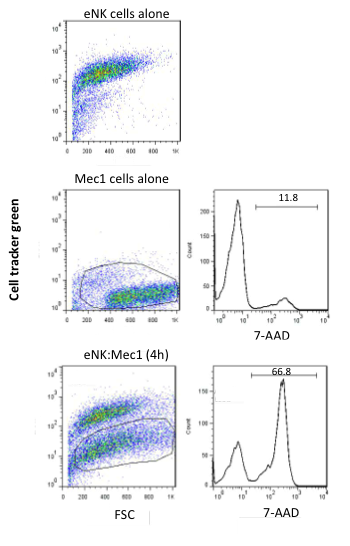
Supplemental Figure 1.** Example of a representative cytotoxicity assay using eNK cells against leukemic Mec1 cells. eNK cells were labeled with Cell Tracker Green (CTG), and this labeling alone is shown in the upper dot plot vs. FSC. Mec1 cells were not labeled with CTG and analyzed at time 0 vs. FSC (middle dot plot). This was used for gating cells and to analyze basal Mec1 cell death on the gated population by 7-AAD staining (middle histogram). eNK cells were mixed at a 1:1 E:T ratio and the cytotoxicity assay developed during 4h. After this time, CTG labeling was analyzed on the mixed population vs. FSC, allowing gating of the Mec1 population (lower left dot plot). This gating was used to analyze cell death induced by eNK cells on Mec1 cells by 7-ADD staining (lower histogram).


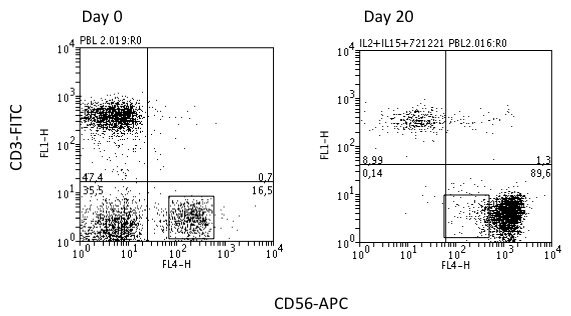


**Supplemental Figure 2.** Example of the variation in the CD56 phenotype in eNK cells (NK3; day 20) with respect to NK cells at day 0; at day 0, most NK cells are CD56^dim^ (squared population), while at day 20, most NK cells are CD56^bright^ and are outside the square.


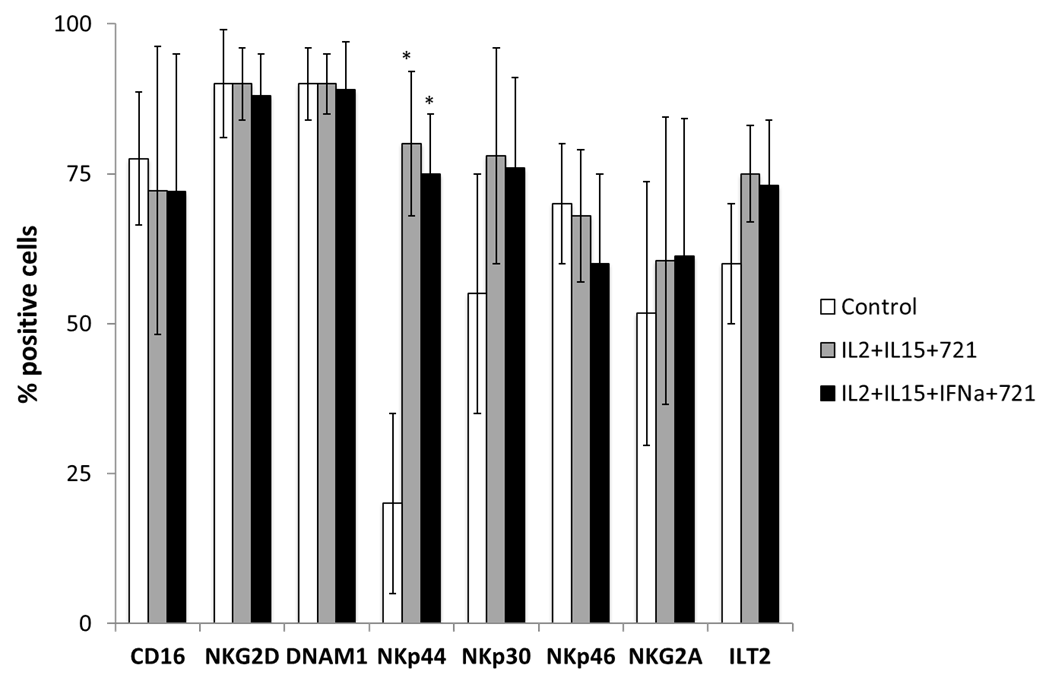
 **Suppl. Figure 3.** Phenotype of expanded NK (eNK) cells. Percentage of NK cells positive for the expression of the indicated surface receptors at day 0 (white bars), and after 20-day expansion following the protocol indicated in Material and Methods in the presence (IL2+IL15+IFNα+721, black bars) or in the absence of IFNα (IL2+IL15+721, grey bars). Data are the mean ± SD of data obtained in cells from the same 10 donors used in the expansion experiments (Supplemental Table I) and in the cytotoxicity assays shown in Fig 2A, except NK5 and NK6. Significance was determined by Student’s t-test; *, *P*<0.05.


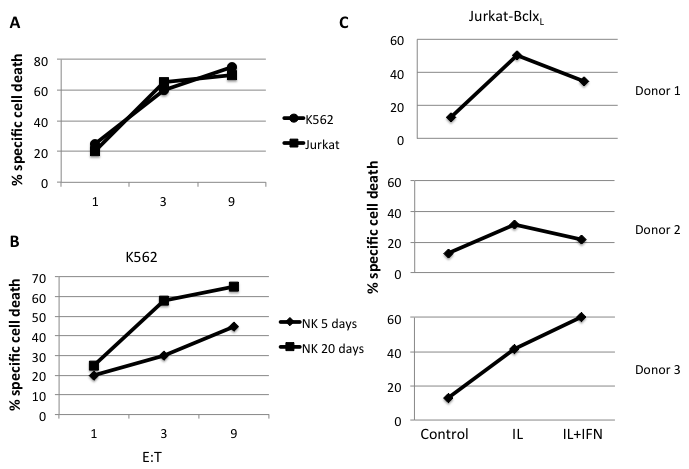
**Supplemental Figure 4.** eNK cells were labeled with cell tracker green (CTG) and tested against different leukemic target cells for 4h at the E:T ratios indicated. Then, target cells were gated as shown in the previous Figure, and cell death was tested by nuclear 7-AAD incorporation. Results are shown as percentage of specific cell death induction, subtracting basal cell death, which was never higher than 15%. A, expanded NK cells were tested at the indicated E:T ratios against HLA-I negative K562 or against HLA-I positive Jurkat cells; B, NK cells activated for 5 or for 20 days, as indicated, with IL-2+IL-15+721.221 feeder cells, were tested on K562 target cells at the indicated E:T ratios; C, non-activated NK cells (control), or eNK cells generated in the presence of IL-2 plus IL-15 (IL) or in the presence of IL-2, IL-15 and IFN-α (IL+IFN) from three different donors were tested at a 5:1 E:T ratio against Jurkat cells over-expressing the anti-apoptotic molecule Bcl-x_L_ (Jurkat-Bcl-x_L_).

**
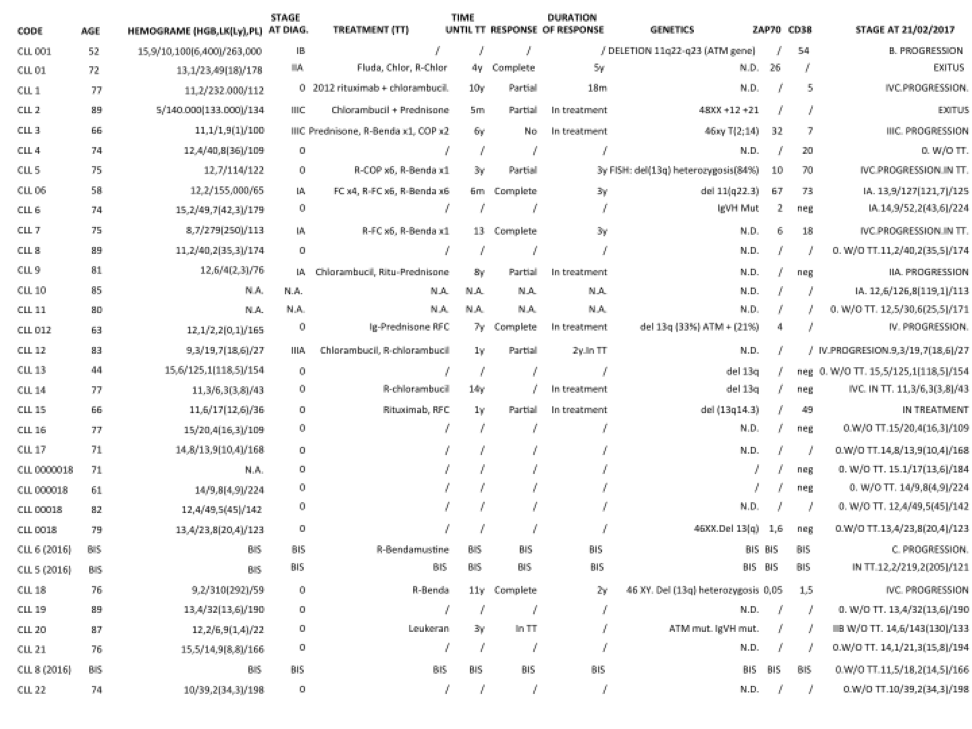
**

**Supplemental Table I (previous page).** Clinical data of the 30 patients enrolled in the study. Hemograme expressed as hemoglobin value in g/dL/number of leukocytes x 10^3^/µL (of which number of lymphocytes x 10^3^/µL)/number of platelets x 10^3^/µL; Fluda,fludarabine; Chlor, chlorambucil; R-Chlor, rituximab plus chlorambucil; R-COP, rituximab plus cyclophosphamide, vincristine and prednisone; R-Benda; rituximab plus bendamustine; FC, fludarabine plus cyclophosphamide; R-FC, rituximab plus fludarabine and cyclophosphamide. Genetics performed: karyotype, deletion of chromosome 13, deletion of chromosome 11q (ATM gene), trisomy of chromosome 12, deletion of chromosome 17 (17p) and IgVH mutation analysis.

|  |  |  | mean |
| --- | --- | --- | --- |
| IL2+IL15+721.221 |  | Donor 1 (NK1) – 34  Donor 2 (NK2) – 32  Donor 3 (NK3) – 33  Donor 4 (NK4) – 42  Donor 5 (NK5) – 30  Donor 6 (NK6) – -  Donor 7 (NK7) – 195  Donor 8 (NK8) - 32  Donor 9 (NK9) - 30  Donor 10 (NK10) - 91 | 58 |
| IL2+IL15+IFNα+721.221 |  | Donor 1 (NK1) – 21  Donor 2 (NK2) – 16  Donor 3 (NK3) – 61  Donor 4 (NK4) – 67  Donor 5 (NK5) – 55  Donor 6 (NK6) – 50  Donor 7 (NK7) – 121  Donor 8 (NK8)- 55  Donor 9 (NK9) - 16  Donor 10 (NK10) - 144 | 61 |

**Supplemental Table II.** Expansion of NK cells following the two protocols tested, expressed as fold expansion with respect to time 0.


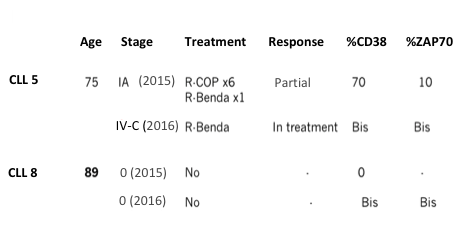


**Supplemental Table III.** Clinical data of patients 5 and 8 at the time of the first test (2015) and at the time of the second test (2016) with eNK cells
